# Src蛋白在肺癌细胞增殖浸润中的作用

**DOI:** 10.3779/j.issn.1009-3419.2011.04.02

**Published:** 2011-04-20

**Authors:** 锐 郑, 晓松 秦, 文洁 李, 健 康

**Affiliations:** 1 110022 沈阳，中国医科大学附属盛京医院第二呼吸内科 2^nd^ Department of Respiratory Internal Medicine, Shengjing Hospital Affliated to China Medical University, Shenyang 110022, China; 2 110004 沈阳，中国医科大学附属盛京医院检验科 Department of Clinical Laboratory, Shengjing Hospital Afiated to China Medical University, Shenyang 110004, China; 3 110001 沈阳，中国医科大学附属第一医院呼吸疾病研究所 Institute of Respiratory Disease, the First Hospital Afiated to China Medical University, Shenyang 110001, China

**Keywords:** 肺肿瘤, Src酪氨酸激酶, 增殖, 浸润, Lung neoplasms, Src tyrosine kinse, Proliferation, Invasion

## Abstract

**背景与目的:**

Src蛋白在肿瘤的发生、发展和转移中具有重要作用。本研究旨在探讨Src蛋白在肺癌细胞中的表达活化情况及其对肺癌细胞增殖浸润的影响。

**方法:**

采用Western blot和免疫沉淀法检测Src蛋白在肺癌细胞株中的表达和磷酸化情况；MT法检测抑制Src酪氨酸激酶活化对肺癌细胞体外增殖的影响；Boyden chamber法检测抑制Src酪氨酸激酶活化对肺癌细胞体外侵袭浸润的影响。

**结果:**

本实验选用的肺癌细胞都存在pp60^src^的表达，非小细胞肺癌（non-small cell lung cancer, NSCLC）细胞株Src蛋白的自主磷酸化水平（SrcpY^418^）明显增高，而人小细胞肺癌细胞株SBC5中几乎检测不到SrcpY^418^。抑制Src酪氨酸激酶活化对肺癌细胞体外增殖呈现出不同的反应，亚微摩尔Src蛋白酪氨酸激酶抑制剂对PC-9和A549细胞体外增殖表现出剂量依赖性抑制作用（*P* < 0.05）；而≤1 μM Src酪氨酸激酶抑制剂对H226、PC14PE6和RERFLCOK细胞增殖没有明显的抑制作用。Src酪氨酸激酶抑制剂对肺癌细胞体外侵袭浸润呈现明显的剂量依赖性抑制作用（*P* < 0.05）。

**结论:**

Src蛋白的活化，而不是过度表达，在NSCLC细胞体外增殖和浸润中发挥着重要作用。

## Abstract (en)

**Background and objective:**

It has been proven that Src played pivotal roles in carcinogenesis, cancer progression and metastasis. The aim of this study is to explore the roles of Src phosphorylation on lung cancer cells.

**Methods:**

Western blot and immunoprecipitation was used to detect the expression and phosphorylation of Src in lung cancer cells. MT and Boyden chamber assay was used to examine the effects of inhibition of Src phosphorylation on proliferation and invasion of lung cancer cells *in vitro*, respectively.

**Results:**

pp60^src^ was expressed in all lung cancer cell lines in this study. All 5 non-small cell lung cancer (NSCLC) cell lines had increased autophosphorylated tyrosine-418, while nearly no phosphorylated Src in small cell lung cancer SBC5 cell line was detected. The effect of inhibition of Src tyrosine kinase on cell proliferation varied among the lung cancer cell lines. Submicromolar Src tyrosine kinase inhibitor (≤1 μM) remarkably suppressed the proliferation of PC-9 and A549 cells in a dose dependent manner (*P* < 0.05), while the same concentration of Src tyrosine kinase inhibitor had no signifcant effect on proliferation of H226, PC14PE6 and RERFLCOK cells. Invasiveness of lung cancer cells was signifcantly suppressed by Src tyrosine kinase in a dose-dependent manner (*P* < 0.05).

**Conclusion:**

Phosphorylation of Src, but not over-expression, plays a pivotal role in proliferation and invasion of NSCLC cell lines *in vitro*.

肺癌是世界范围肿瘤相关性死亡的主要病因，近年来肺癌药物治疗的新策略转向抑制肿瘤生长进展中特异性通路和关键分子的分子靶向治疗^[1]^。其中，表皮生长因子受体（epidermal growth factor receptor, EGFR）酪氨酸激酶抑制剂吉非替尼和厄洛替尼已成功地用于治疗某些晚期非小细胞肺癌（non-small cell lung cancer, NSCLC）患者，然而也只是部分腺癌患者更有效^[1, 2]^。因此，有必要加强对其它重要的靶分子的研究。

*Src*是最早报道的癌基因，反转录病毒基因*V-Src*具有致癌性，其同源的原癌基因*C-Src*是人或动物细胞中的正常基因，它们编码相对分子质量为6×10^4^的非受体蛋白酪氨酸激酶^[3]^。Src蛋白质产物由N端、Src同源域、激酶域和C端组成，Src蛋白在调控细胞的生存、增生、黏附、运动和细胞信号转导等方面发挥重要作用^[4]^。人类很多肿瘤都存在Src蛋白的过度表达和/或活化，Src蛋白在肿瘤的发生、发展和转移中具有重要作用^[5, 6]^。近来研究发现Src蛋白在肺癌中也有过度表达和/或活化^[6-8]^，体外实验和临床研究^[6, 7]^表明抑制Src酪氨酸激酶对肺癌患者有效，然而其在肺癌进展中的作用及机制尚不清楚，本文研究Src蛋白在6种不同类型的肺癌细胞中的表达和活化，进而探讨Src蛋白在肺癌细胞增殖浸润中的作用。

## 1 材料与方法

### 1.1 主要试剂、药品和设备

人支气管上皮细胞BEAS2B，人肺腺癌A549细胞和RERFLCOK细胞购于美国模式培养物保藏所；人肺腺癌PC-9细胞和小细胞肺癌SBC5细胞购于日本IBL公司；人肺腺癌PC14PE6细胞和鳞癌H226细胞为美国德州大学安德森癌症中心所赠。选择性Src酪氨酸激酶抑制剂4-苯胺喹唑啉衍生物由英国AstraZeneca公司提供。鼠抗Src单克隆抗体购自Upstate Biotechnogy公司。兔抗Src（酪氨酸418）磷酸特异性多克隆抗体购自Biosource International公司。增强型化学发光（enhanced chemiluminescence ECL）试剂盒购自Amersham Pharmacia Biotech公司。溴化二甲噻唑二苯四氮唑[3-(4, 5-dimethylthiazol-2-yl)-2, 5 diphenyl tetrazolium, MTT]购自美国Sigma公司。胶原Ⅰ购自BD Biosciences公司。纤维联结蛋白：日本岩城硝子公司。

### 1.2 细胞培养

BEAS2B细胞在含10%小牛血清的LHC9/ RPMI-1640培养基中，A549细胞、PC-9细胞、H226细胞和RERFLCOK细胞在含10%小牛血清的DMEM培养基中，PC14PE6细胞和SBC5细胞在含10%小牛血清的RPMI-1640培养基中，于37 ℃、5%CO_2_饱和湿度培养。每毫升培养液含100 U青霉素、100 µg链霉素。

### 1.3 Western blot和免疫沉淀法检测Src蛋白在肺癌细胞中的表达和活化

首先提取总蛋白，将43-45代BEAS2B细胞以及对数生长期的6种肺癌细胞接种于10 cm培养皿上，待细胞长满培养皿的80%时，用PBS洗两次，加入细胞裂解缓冲液，于干冰上速冻。待细胞溶解后，用塑料刮匙收集裂解液，低温超声粉碎，离心20 min（16, 000 r/min, 0 ℃），用Bradford蛋白分析试剂测量所提取蛋白的浓度。

对于免疫沉淀，取500 μg蛋白上清用鼠抗Src单克隆抗体孵育过夜（4 ℃），然后用蛋白G-琼脂糖珠在旋转台上孵育2 h（4 ℃），用溶解缓冲液反复清洗4次。取100 μg蛋白或之前清洗过的免疫沉淀物进行SDS聚丙烯酰胺凝胶电泳，然后转移蛋白到硝酸纤维素膜上，于5%的小牛血清白蛋白缓冲液中阻断1 h，用鼠抗Src单克隆抗体或兔抗Src（酪氨酸418）磷酸特异性多克隆抗体孵育过夜（4 ℃），接下来于物种特异性的辣根过氧化物酶链接的二抗中孵育1 h，洗涤用ECL显示免疫反应条带，用Image J软件进行图像分析处理，计算灰度值及相对强度。

### 1.4 Western blot和免疫沉淀法检测Src酪氨酸激酶抑制剂对Src蛋白在肺癌细胞中的表达和活化的影响

将各种肺癌细胞接种于6孔板上，待细胞长满培养皿的80%时，给予不同浓度的Src酪氨酸激酶抑制剂处理60 min，然后提取总蛋白，进行免疫沉淀和Western blot，具体步骤同上。由于Src酪氨酸激酶抑制剂对Src蛋白在肺癌细胞中的表达没有明显影响，我们把Src蛋白在肺癌细胞中的表达作为内对照。

### 1.5 MTT法检测Src酪氨酸激酶抑制剂对肺癌细胞体外增殖的抑制作用

肺癌细胞（PC-9、A549、H226、PE14PE6和RERFLCOK）2×10^4^/mL接种于96孔板，37 ℃、5%CO_2_培养箱孵育24 h后，每孔加入不同浓度的Src酪氨酸激酶抑制剂（0.01 μM、0.03 μM、0.1 μM、0.3 μM和1 μM）0.1 mL，设置细胞对照及空白对照，每组样本设6个复孔，置37 ℃、5%CO_2_培养箱孵育72 h后，每孔加入50 μL MTT（2 mg/mL），继续培养2 h，去掉培养液，加入100 μL DMSO，充分振荡溶解深蓝色结晶，于MTP-120微孔板酶免比色仪下测定吸光度值，550 nm和630 nm分别为检测和参考波长。最终计算3次实验的平均值^[9]^。

### 1.6 Boyden chamber方法检测Src酪氨酸激酶抑制剂对肺癌细胞侵袭浸润的抑制作用^[9]^

使用胶原Ⅰ（30 μg/filter）包埋孔径为8 μm的transwell chambers。血清饥饿24 h的肺癌细胞（1×10^5^/200 μL）悬浮于含有不同浓度的Src酪氨酸激酶抑制剂（0.01 μM、0.03 μM、0.1 μM、0.3 μM和1 μM）的培养液中，加到槽的上层。含有10 μg/mL纤维联结蛋白的趋化液加到槽的下层。置37 ℃、5%CO_2_培养箱孵育6 h后，用棉棒擦掉没有浸润到下层的细胞，切下分隔膜，使用Diff-Quik系统进行固定和染色。在200×亮视野显微镜下随机选取6个视野计数细胞数，取3个独立实验的平均值进行统计学分析。在进行侵袭浸润实验的同时，用MTT法检测Src酪氨酸激酶抑制剂对细胞生存活力的影响。

### 1.7 统计学方法

采用SPSS 11.0统计分析软件进行方差分析，*P* < 0.05为有统计学差异。

## 2 结果

### 2.1 Src蛋白在肺癌细胞中的表达和磷酸化

#### 2.1.1 Src蛋白在肺癌细胞中的表达

研究发现人正常支气管上皮细胞BEAS2B，人NSCLC细胞（PC-9、A549、PC14PE6、RERFLCOK和H226）和人小细胞肺癌SBC5细胞中都存在pp60^src^的表达。pp60^src^在A549细胞中的表达水平略高于BEAS2B细胞中的表达，在PC-9细胞中的表达和BEAS2B细胞接近，而在其余的人肺腺癌、鳞癌和小细胞肺癌细胞中的表达均明显低于BEAS2B细胞。pp60^src^在人小细胞肺癌SBC5细胞中的表达水平约为人正常支气管上皮细胞BEAS2B的38%（图 1）。

**1:**
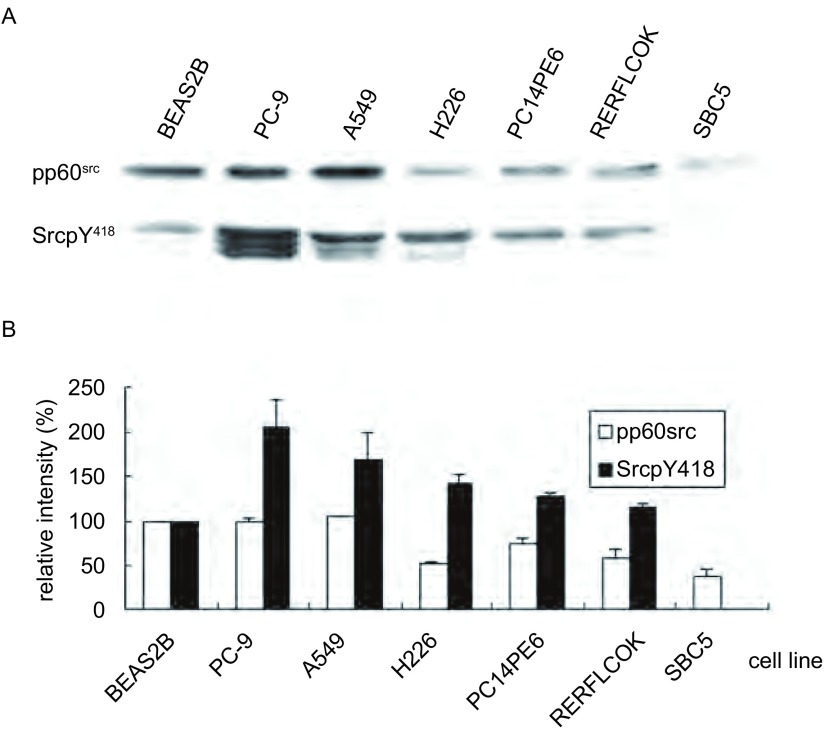
肺癌细胞中Src蛋白的表达和磷酸化。A：肺癌细胞中Src蛋白的表达和磷酸化的Western blot检测结果；B：肺癌细胞中Src蛋白的表达和磷酸化的分析图。 Expression and phosphorylation of Src in lung cancer cells. A: Expression and phosphorylation of Src in lung cancer cells by Western blot; B: The analysis of expression and phosphorylation of Src in lung cancer cells.

#### 2.1.2 Src蛋白在肺癌细胞中的磷酸化

BEAS2B细胞中Src蛋白呈现弱的自主磷酸化（SrcpY^418^），NSCLC细胞中Src蛋白的自主磷酸化水平明显增高，PC-9细胞中的SrcpY^418^水平为人正常支气管上皮细胞BEAS2B中的2.1倍；A549细胞中SrcpY^418^水平为BEAS2B中的1.7倍；H226、PC14PE6和RERFLCOK中SrcpY^418^水平也分别为正常的1.4、1.3和1.2倍。然而在人小细胞肺癌SBC5细胞中几乎检测不到SrcpY^418^水平（图 1）。

### 2.2 Src酪氨酸激酶抑制剂对NSCLC细胞中Src蛋白表达和磷酸化的作用

Src酪氨酸激酶抑制剂对NSCLC细胞中pp60^src^的表达没有抑制作用，pp60^src^作为内对照。RERFLCOK、H226和PC14PE6细胞对Src酪氨酸激酶抑制剂不敏感，≤1 μM Src酪氨酸激酶抑制剂对RERFLCOK细胞中SrcpY^418^水平几乎无影响，≥0.3 μM的Src酪氨酸激酶抑制剂才能够抑制H226和PC14PE6细胞中SrcpY^418^水平。而0.03 μM Src酪氨酸激酶抑制剂使PC-9和A549细胞Src蛋白的自主磷酸化（SrcpY^418^）降低50%以上，Src酪氨酸激酶抑制剂对PC-9和A549细胞SrcpY^418^呈现明显的剂量依赖的抑制作用（图 2）。

**2:**
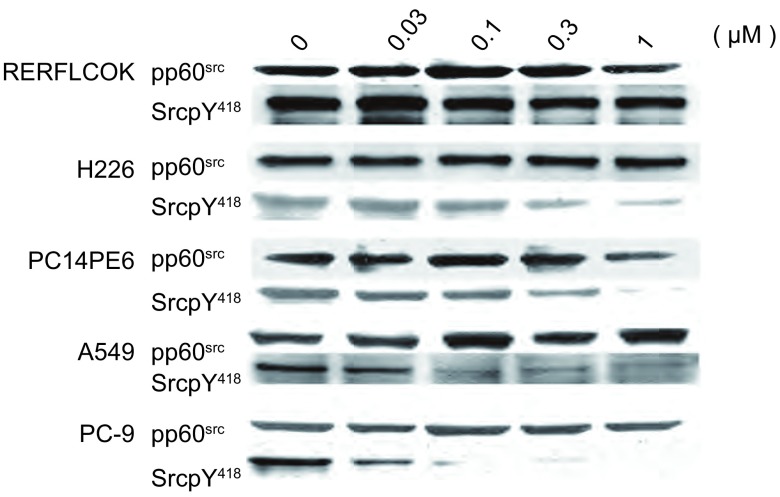
Src酪氨酸激酶抑制剂对NSCLC细胞中Src蛋白表达和磷酸化的作用 Effect of Src tyrosine kinase inhibitor on Src expression and phosphorylation in nonsmall cell lung cancer (NSCLC) cells

### 2.3 Src酪氨酸激酶抑制剂对NSCLC细胞增殖的抑制作用

0.01 μM-1 μM 5种浓度的Src酪氨酸激酶抑制剂作用于NSCLC细胞72 h后，MTT法检测Src酪氨酸激酶抑制剂对NSCLC细胞体外增殖的抑制作用。Src酪氨酸激酶抑制剂对5种NSCLC细胞体外增殖呈现出不同的反应。≤1 μM的Src酪氨酸激酶抑制剂对H226、PC14PE6和RERFLCOK细胞增殖没有明显的抑制作用。Src酪氨酸激酶抑制剂对PC-9和A549细胞表现出明显的剂量依赖性抑制作用。0.1 μM、0.3 μM和1 μM的Src酪氨酸激酶抑制剂对PC-9细胞和A549细胞增殖的抑制率分别为53.6%（*P* < 0.001）、72.2%（*P* < 0.001）和82.4%（*P* < 0.001）；以及30.3%（*P* < 0.05）、38.5%（*P* < 0.001）和46.6%（*P* < 0.001）（图 3）。

**3:**
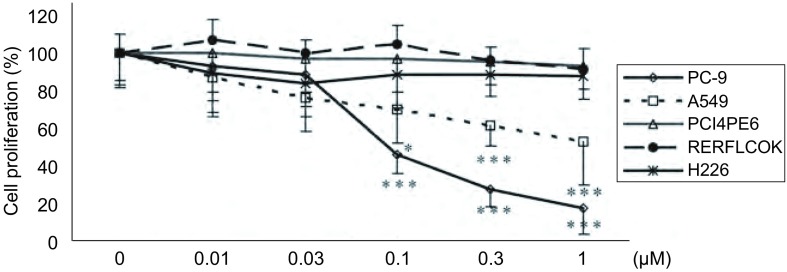
Src酪氨酸激酶抑制剂对NSCLC细胞体外增生的影响。与对照组相比，^*^P < 0.05，^***^P < 0.001。 Effect of Src tyrosine kinase inhibitor on NSCLC cell proliferation. ^*^P < 0.05, ^***^P < 0.001 *vs* control group.

### 2.4 Src酪氨酸激酶抑制剂对NSCLC细胞体外侵袭浸润的影响

Src酪氨酸激酶抑制剂对NSCLC细胞呈现明显的剂量依赖性抑制作用。特别是PC-9细胞对Src酪氨酸激酶抑制剂非常敏感，0.03 μM、0.1 μM、0.3 μM和1 μM Src酪氨酸激酶抑制剂对PC-9细胞体外侵袭浸润的抑制率分别为17.3%（*P* < 0.05）、63.5%（*P* < 0.001）、82.7%（*P* < 0.001）和91.7%（*P* < 0.001）。0.3 μM和1 μM Src酪氨酸激酶抑制剂对A549细胞体外侵袭浸润的抑制率分别为36.4%（*P* < 0.001）和77.9%（*P* < 0.001）。1 μM Src酪氨酸激酶抑制剂对PC14PE6细胞和H226细胞体外侵袭浸润也表现出明显的抑制作用，抑制率分别为54.7%（*P* < 0.001）和36.9%（*P* < 0.001）。同时进行的MTT分析说明侵袭浸润实验所用的药物浓度和时间对NSCLC细胞活力无明显影响（图 4，图 5）。

**4:**
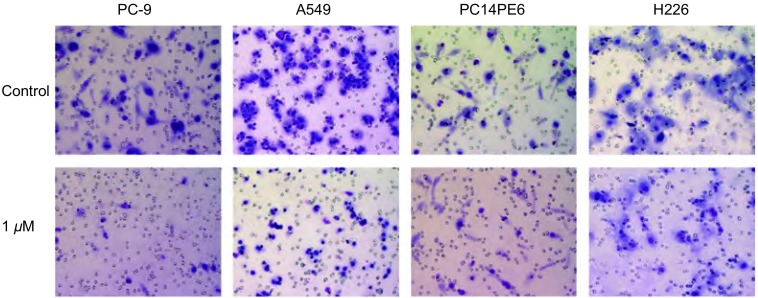
Src酪氨酸激酶抑制剂对NSCLC细胞体外侵袭浸润的影响 Effect of Src tyrosine kinase inhibitor on NSCLC cell invasiveness

**5:**
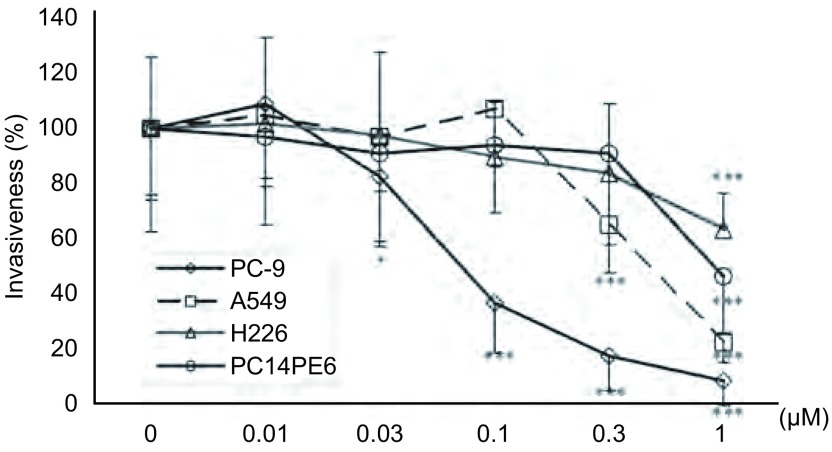
Src酪氨酸激酶抑制剂对NSCLC细胞体外侵袭浸润的影响。与对照组相比，^*^P < 0.05，^***^P < 0.001。 Effect of Src tyrosine kinase inhibitor on NSCLC cell invasiveness. ^*^P < 0.05, ^***^P < 0.001 *vs* control group.

## 3 讨论

人类很多肿瘤都涉及到原癌基因的异常表达和活化。虽然大多数原癌基因产物的生物化学功能还不是很清楚，它们当中的一些原癌基因产物具有蛋白酪氨酸激酶活性，其中pp60src是研究最广泛的蛋白激酶。肺癌、乳腺癌、结肠癌和胰腺癌等很多肿瘤都存在Src蛋白的过度表达和/或活化^[5, 10]^。

本研究发现，NSCLC中Src蛋白的活化和Src蛋白的表达不同步。本实验所用的5种NSCLC细胞都存在Src蛋白的表达和活化，但是Src蛋白的表达水平无明显增加，只有肺腺癌A549细胞的Src蛋白水平略高于正常支气管上皮细胞中的水平，肺腺癌PC-9细胞中的Src蛋白水平与正常接近，而其余的肺腺癌细胞PC14PE6、RERFLCOK和肺鳞癌H226中Src蛋白的表达都明显低于正常水平。与之相反的是，NSCLC中Src蛋白的自主磷酸化（SrcpY^418^）水平都高于正常支气管上皮细胞，特别是腺癌中Src蛋白的自主磷酸化水平明显高于正常。这与Masaki等^[8]^学者的研究结果是一致的。此外，Rosen等^[11]^也发现和正常乳腺组织相比，乳腺肿瘤的Src激酶活性明显增高，而Src蛋白的表达相对正常。因此可以推测，在NSCLC中，Src激酶活性增高不是或不完全由于Src蛋白表达的增加。Src蛋白的活化，而不是过度表达，可能在NSCLC进展过程中发挥更重要的作用。

NSCLC中Src蛋白活化的方式是多种多样的^[6, 7]^。本研究发现，人NSCLC中正常调节自主磷酸化位点酪氨酸418（SrcpY^418^）的直接磷酸化明显增高。另有报道^[12]^，肺腺癌中存在Src蛋白酪氨酸530的解磷酸化。此外，Src作为胞浆内蛋白，能与细胞膜上的许多生长因子受体相互作用，进而激活Src蛋白^[6]^。激活的Src蛋白使其下游信号发生瀑布效应，通过组织丝裂原活化的蛋白激酶（mitogen activated protein kinase, MAPK）以及信号传导和转录激活因子3（signal transducers and activators of transcription 3, STAT3）信号通路促进肿瘤细胞增殖和转移；通过血管内皮生长因子（vascular endothelial growth factor, VEGF）和上皮基质转化（epithelial-mesenchymal transition, EMT）促进肿瘤新生血管形成、浸润和转移^[13]^。研究^[8]^表明，肺腺癌中Src蛋白活化程度和腺癌肿块的大小相关，说明Src蛋白的活化能促进肺腺癌增殖和进展。

前期的研究^[9, 14]^发现，抑制Src酪氨酸激酶自主磷酸化，能够抑制肺腺癌细胞的体内外增殖和体外浸润。在此基础上，本研究进一步探讨了Src酪氨酸激酶抑制剂对5种不同的NSCLC细胞的作用。结果表明，亚微摩尔Src酪氨酸激酶抑制剂抑制Src自主磷酸化，灭活Src蛋白，能够明显抑制Src蛋白自主磷酸化水平较高的肺腺癌PC-9和A549细胞的体外增殖和浸润。1 μM Src酪氨酸激酶抑制剂对PC14PE6和H226细胞的游走浸润也有明显的抑制作用，而1 μM Src酪氨酸激酶抑制剂对PC14PE6、H226和RERFLCOK细胞的增殖则没有明显的抑制作用。因此，活化的Src蛋白在NSCLC细胞体外增殖和浸润中发挥着重要作用，同一肿瘤的不同类型，Src蛋白对细胞增殖和浸润的影响可能是不同的^[9]^，Src蛋白自主磷酸化水平较高的NSCLC细胞可能对Src酪氨酸激酶抑制剂更敏感。另一方面，抑制NSCLC体外侵袭浸润所需的浓度明显低于抑制细胞增殖所需的浓度，提示Src蛋白调节细胞增殖和浸润的方式以及信号传导通路可能是不同的^[9]^。

与NSCLC相反，本研究观察到小细胞肺癌SBC5细胞中Src蛋白的表达和活化程度都很低，Src蛋白的表达水平仅为正常支气管上皮细胞的38%，而SrcpY^418^检测不出。因此，与Src蛋白在NSCLC，特别是腺癌中的作用相比，Src蛋白在小细胞肺癌进展中的作用有限，这还有待深入研究。

综上所述，Src蛋白的活化，而不是过度表达，在NSCLC细胞体外增殖和浸润中发挥着重要作用，其机制有待进一步探讨。Src酪氨酸激酶抑制剂可以选择性用于Src蛋白高度活化的NSCLC的分子靶向治疗。
